# Can physicians’ judgments of futility be accepted by patients?: A comparative survey of Japanese physicians and laypeople

**DOI:** 10.1186/1472-6939-13-7

**Published:** 2012-04-20

**Authors:** Yasuhiro Kadooka, Atsushi Asai, Seiji Bito

**Affiliations:** 1 Department of Bioethics, Kumamoto University Graduate School of Medical Science, 1-1-1 Honjo, Kumamoto City, Kumamoto 860-8556, Japan; 2 Division of Clinical Epidemiology and division of postgraduate clinical training center, National Hospital Organization Tokyo Medical Center Clinical Research Center, Tokyo, Japan

## Abstract

**Back ground:**

Empirical surveys about medical futility are scarce relative to its theoretical assumptions. We aimed to evaluate the difference of attitudes between laypeople and physicians towards the issue.

**Methods:**

A questionnaire survey was designed. Japanese laypeople (via Internet) and physicians with various specialties (via paper-and-pencil questionnaire) were asked about whether they would provide potentially futile treatments for end-of-life patients in vignettes, important factors for judging a certain treatment futile, and threshold of quantitative futility which reflects the numerical probability that an act will produce the desired physiological effect. Also, the physicians were asked about their practical frequency and important reasons for futile treatments.

**Results:**

1134 laypeople and 401 (80%) physicians responded. In all vignettes, the laypeople were more affirmative in providing treatments in question significantly. As the factors for judging futility, medical information and quality of life (QOL) of the patient were rather stressed by the physicians. Treatment wish of the family of the patient and psychological impact on patient side due to the treatment were rather stressed by laypeople. There were wide variations in the threshold of judging quantitative futility in both groups. 88.3% of the physicians had practical experience of providing futile treatment. Important reasons for it were communication problem with patient side and lack of systems regarding futility or foregoing such treatment.

**Conclusion:**

Laypeople are more supportive of providing potentially futile treatments than physicians. The difference is explained by the importance of medical information, the patient family’s influence to decision-making and QOL of the patient. The threshold of qualitative futility is suggested to be arbitrary.

## Background

The issue of medical futility has been debated for some time, but there has been little consensus leading to a resolution [1-4]. The discussion has been based primarily on theoretical assumptions and empirical surveys are relatively scarce [5]. Among these empirical surveys, few have focused on the patient perspective. The core issue is disagreement between healthcare professionals and patients about providing or forgoing futile treatments. The importance of communication and negotiation by both sides has been emphasized [3,6]. Balancing the contrasting viewpoints requires finding common ground [7], and exploring potential benefits of an intervention beyond those normally considered by medical teams becomes mandatory [8]. Therefore, we think that evaluation of laypeoples’ attitudes is essential for understanding the issue.

Two major developments led to recognition of medical futility as an issue: the rise of patient autonomy and advances in medical technology [4]. In Japan, respect for patient autonomy has been emphasized in clinical practice along with introduction of Western bioethics concepts [9]. Universal health insurance coverage ensures provision of high-tech treatment for almost all Japanese. Under such conditions, Japan has the highest life expectancy in the world, though there are concerns of healthcare expenditure inflation, overzealous treatments and frequent medical examinations. Public interest in end-of-life care is very high, and most healthcare workers experience difficulty and questions regarding it [10].

Surveys and discussions about medical futility have been scarce in Japan. Given the lack of precedent and laws to address it, such treatments are frequently provided to patients [11-13]. Yet, several guidelines directed to end-of-life care have been developed in recent years that include mention of meaningless and non-beneficial life-sustaining treatments and forgoing them [14,15]. Although these guidelines fail to explicitly address the futility issue, and fail to provide healthcare workers with the authorization to judge and forego life-sustaining treatments, it implies that this issue is at the forefront in Japanese clinical settings.

Bagheri and colleagues conducted a questionnaire survey of Japanese bioethics experts and concluded that medical futility is especially relevant to the Japanese healthcare system [5]. Although this was the first survey regarding medical futility in Japan, the small sample size and bioethics expert respondents (who are likely influenced by Western concepts) may have led to results that fail to accurately reflect the views of the Japanese population as a whole. In 2009, we conducted a preliminary interview survey involving Japanese physicians and nurses, and inquired about actual cases in which they judged providing treatments to be futile, and explored their views on futility [11]. All participants had provided such treatments in the past, and provided details on patient conditions, treatments provided, reasons for providing the treatments, and factors they used to judge a certain treatment futile. Most cases involved end-stage or incompetent patients. In addition to treatment requests from patients, such treatments were provided due to issues related to the decision-making process, the patient-healthcare worker relationship, and the lack of standards for forgoing treatments and judging certain treatments futile. Factors for judging a certain treatment futile included medical factors and patient condition, social norms, and cost-benefit considerations. Based on findings from that survey, we designed a questionnaire survey directed at Japanese laypeople and physicians to evaluate differences in attitudes regarding medical futility, including whether to provide potentially futile treatments and factors for judging a certain treatment futile at the bedside level.

## Methods

### Study design

We performed a cross-sectional study using an anonymous questionnaire survey. All participants were asked identical questions. The present study was approved by the ethics committee at Kumamoto University Graduate School of Medical Science in July 2010.

### Participants

To sample physician-respondents, we contacted 42 physicians who practice at the forefront of Japanese healthcare settings one-by-one. All consented and distributed eleven-page questionnaires to co-workers (physicians) at their facilities. A total of 500 questionnaires were distributed at 53 medical facilities of 11 prefectures in Japan. Meanwhile, we commissioned a reputable market researcher (Cross Marketing Inc. http://www.cross-m.co.jp/) to sample layperson-respondents. The inclusion criteria were: non-healthcare worker, Japanese nationality, living in Japan, able to understand Japanese, and 20–69 years old. Participants were chosen from 1.35 million registered panelists, who accessed and answered the questionnaire via the Internet. All questionnaires were sent in August 2010.

### Questionnaire

The questionnaire was developed based on findings of our preliminary study [11] and consisted primarily of three sections. The first section asked about demographic and professional characteristics of participants. In the second section, we asked about attitudes toward three hypothetical vignettes involving potentially futile treatments that reflect conditions of Japanese end-of-life care in recent years: malignant neoplasm is the most frequent cause of death, the issue of whether brain death is equivalent to the final death of a person was raised during the revision of the Organ Transplant Law, and the impact of the increase in demented elderly patients due to the rapidly aging population on healthcare planning. The vignettes are summarized in Table 1. Respondents were asked whether to provide the treatment in question and rate the importance of the patient’s medical condition for providing the treatment using 5-point Likert scale answers. The third section offered 20 items for judging a certain treatment futile and asked participants to select three items they considered especially important (Table 2). We also asked about the threshold of quantitative futility which reflects the numerical probability that an act will produce the desired physiological effect: “If the likelihood of success is under ( )%, I would judge the treatment futile.” Participants who could not identify a numerical value selected the option “I do not know.” In an additional section, only physician-respondents were asked about frequency and reasons for providing treatments judged futile (Table 3).

**Table 1:** Attitudes to hypothetical cases (number of respondents who answered “YES”)


**Vignette 1 :** A PATIENT WITH ADVANCED LUNG CANCER REQUESTS ANOTHER COURSE OF CHEMOTHERAPY WITH A STRONG-WILLED, “I NEVER GIVE UP TO THE END.” THE PATIENT RECEIVED PREVIOUS COURSES OF CHEMOTHERAPY REPEATEDLY AND DETERIORATED SEVERELY. HIS DOCTORS ESTIMATED THAT THERE WERE NO LONGER ANY AGENTS ANTICIPATED EFFECTIVE FOR HIS CONDITION, AND HIS LIFE EXPECTANCY MAY BE SEVERAL WEEKS.		
	**Physicians (%)**	**Laypeople (%)**
Q: Should another course of chemotherapy be administered?*	131 (33.2)	742 (65.4)
Q: Are his doctors’ professional opinions important for judging whether to administer it?*	306 (77.5)	506 (44.6)
**Vignette 2 :** A 21-YEAR-OLD PATIENT DIAGNOSED AS BRAIN DEAD DEVELOPS RENAL FAILURE AND SEVERE SYSTEMATIC EDEMA. PARENTS OF THE PATIENT UNDERSTAND THE CONDITION OF THEIR SON BUT REQUEST BLOOD PURIFICATION THERAPY SAYING, "WE DO NOT WANT TO LOSE OUR SON YET. WE HOPE TO PROVIDE UNLIMITED TREATMENT." THE PATIENT HAS NO ADVANCE DIRECTIVES REGARDING HIS MEDICAL TREATMENT.		
	**Physicians (%)**	**Laypeople (%)**
Q: Should blood purification therapy be provided?*	97 (24.4)	490 (43.2)
Q: Is the patient’s brain death important for judging whether to provide the therapy?*	323 (81.4)	611 (53.9)
**Vignette 3 :** A VERY OLD WOMEN WHO IS INCOMPETENT DUE TO ADVANCED DEMENTIA AND BEDRIDDEN FOR A DAY IS DIAGNOSED WITH GASTRIC CANCER. THE MEDICALLY OPTIMAL TREATMENT FOR THE CANCER STAGE IS GASTRIC RESECTION, WHICH IS EXPECTED TO PROLONG HER LIFE FOR ONE YEAR. SHE HAS NO ADVANCE DIRECTIVES ABOUT MEDICAL TREATMENTS.		
	**Physicians (%)**	**Laypeople (%)**
Q: Should gastric surgery be performed?*	59 (14.8)	395 (34.8)
Q: Is her advanced dementia important for judging whether to perform gastric resection?*	309 (77.6)	474 (41.8)

**Table 2:** Factors for judging a certain treatment futile

**Factor**		**Physicians**	**Laypeople**
		**n = 1161 (%)**	**n = 3330 (%)**
1	Request for the treatment from the patient	43.4	69.4
2	Request for the treatment from the patient’s family	6.7	26.4
3	Lack of patient’s wish for the treatment	34.7	28.0
4	Lack of patient’s family’s wish for the treatment	6.0	4.3
5	Cognitive function of the patient	6.2	4.8
6	Patient age	6.0	8.0
7	Patient being close to death	19.7	14.3
8	Therapeutic effect for prolongation of the patient’s life	15.0	17.7
9	Therapeutic effect for QOL (quality of life) of the patient	40.6	20.0
10	Religion of the patient	3.5	1.3
11	Value judgment of the patient about treatment course	7.2	7.8
12	Psychological impact of the treatment on the patient	4.0	14.0
13	Psychological impact of the treatment on the patient’s family	0.7	3.4
14	Maintenance of the patient-healthcare worker relationship	2.0	1.5
15	Likelihood of recovery or cure due to the treatment	41.6	35.3
16	Physical harm (side effect or complication) caused by the treatment	16.2	10.1
17	Theoretical medical appropriateness of providing the treatment	28.4	14.2
18	Value judgment of the healthcare worker about treatment course	2.2	3.1
19	Psychological impact on the healthcare workers	1.0	1.7
20	Cost-benefit balance of the treatment	4.2	8.5

Each respondent chose three items that he/she considered especially important for judging futility.

**Table 3:** Reasons for providing treatments which participants judged futile

**Reason**		**n = 1144 (%)**
1	Request for the treatment from the patient	66.6
2	Request for the treatment from the patient’s family	32.4
3	Lack of refusal of the patient for the treatment	7.2
4	Lack of refusal of the patient’s family for the treatment	2.0
5	Healthcare worker’s inadequate explanation about futility of the treatment	16.2
6	Patient’s insufficient understanding about futility of the treatment	21.4
7	To satisfy the patient	37.7
8	To satisfy the patient’s family	10.7
9	Maintenance of the patient-physician relationship	10.2
10	Maintenance of the relationship between the patient’s family and physician	2.7
11	Request or instruction of another physician regarding the treatment	4.2
12	Feeling sorry for the patient	1.2
13	Lack of public standards about judging futility	23.2
14	Lack of public standards about forgoing treatments	19.2
15	Professional attitude to do everything as much as possible	9.7
16	Avoidance of legal issue	16.7
17	Commercial management of the medical facility	3.7

Each respondent chose three items that he/she considered an especially important reason for providing treatments judged futile.

### Statistical analysis

The 5-point Likert scale answers were divided into two categories: YES (“I definitely think so” and “I think so”) and NO (“I am unsure,” “I somewhat don’t think so,” and “I don’t think so”). (We assumed that physician-respondents who answered “I am unsure” would not recommend the medical intervention in question in the vignettes and that layperson-respondents who answered it would not consent to the treatment. Therefore we categorized the response “I am unsure” as “NO”.) Differences in proportions among independent categorical variables were tested using the chi-square test and numerical variables were compared using the unpaired t-test. A logistic regression model was used when univariate analysis revealed statistically significant relations between independent variables (age, sex, religious belief, and experience of feeling inappropriate medical treatment) and respondents’ answers. A *P*-value of less than 0.05 was considered significant. All statistical analyses were conducted using SPSS (version 18.0).

## Results

### Study participants

We received 401 questionnaires from physician-respondents by mail (response rate was 80.2%) and 1134 completed surveys from laypeople via the Internet (16190 were accessed within three days). Respondent characteristics are shown in Table 4. There were significant differences between groups in age, sex, religious belief, and experience of impression of practicing inappropriate medical treatment. The average period of professional practice was 15.6 years. The majority were internists (47.9%), followed by surgeons (31.7%) and other specialties (18.5%), with 85% of physicians working at hospitals. Almost half of the laypeople consulted physicians several times a year, and most frequently consulted at clinics. Approximately 87% of physicians had practical experience with end-of-life care, while 68.6% of laypeople experienced the death of a close person in the last ten years.

**Table 4:** Respondent characteristics

	**Physicians**	**Laypeople**
	**(n = 401)**	**(n = 1134)**
Age*		
Mean (years)	41.6	44.5
Range	24–87	20–69
Sex*		
Male (%)	314 (78.3)	578 (51.0)
Female (%)	82 (20.4)	556 (49.0)
No answer	5	0
Religious belief*		
Yes (%)	53 (13.2)	92 (8.1)
No (%)	343 (85.5)	1016 (89.6)
No answer (%)	5 (0.12)	26 (2.3)
Experienced impressions of practicing inappropriate medical treatments*		
Yes (%)	334 (83.3)	460 (40.6)
No (%)	60 (15.0)	674 (59.4)
No answer (%)	7 (1.7)	0
Mean years of practice (range)	15.6 (1–67)	−
Specialty (%)		−
Internal medicine	192 (47.9)	
Surgical medicine	127 (31.7)	
Other	74 (18.5)	
No answer	8 (2.0)	
Affiliated facility (%)		−
Clinic	17 (4.2)	
Private hospital	151(37.7)	
Public hospital	121 (30.2)	
University hospital	66 (16.5)	
Other	42 (10.5)	
No answer	4 (1.0)	
Practical experience with end-of-life stage patients		
Yes (%)	348 (86.8)	
No (%)	48 (12.0)	
No answer (%)	5 (1.2)	
Frequency of consulting with medical doctors	−	
Almost every day (%)		3 (0.3)
Several times a week (%)		20 (1.8)
Several times a month (%)		272 (24.0)
Several times a year (%)		563 (49.6)
Never (%)		276 (24.3)
Most frequent medical facility of consult	−	
Clinic (%)		576 (50.8)
Private hospital (%)		226 (19.4)
Public hospital (%)		55 (4.9)
University hospital (%)		217 (19.1)
Other (%)		66 (5.8)
Experienced death of a close person in the last 10 years	−	
Yes (%)		778 (68.6)
No (%)		356 (31.4)

* *P* < 0.05.

### Attitudes toward treatments in hypothetical vignettes

In all vignettes, laypeople were significantly more likely to answer in the affirmative than physicians for providing treatments in question (Table 1). The logistic regression model revealed that age was the only significant independent factor in all cases (*p* = 0.000, 0.000 and 0.016, respectively). Younger respondents tended to support providing the treatments in question.

### Factors for judging a certain treatment futile

Respondents chose three items from the list (Table 2). Items regarding the patient’s preference for the treatment (1, 3) and “Likelihood of recovery or cure due to the treatment” (15) were stressed in both groups. “Therapeutic effect for QOL (quality of life) of the patient” (9), “Physical harm (side effect or complication) of the treatment” (16), “Theoretical medical appropriateness of providing the treatment” (17), and “Patient being close to death” (7) were stressed by the physicians. On the contrary, “Request for the treatment from the patient’s family” (2) and “Psychological impact of providing the treatment on the patient” (12) were stressed by the laypeople.

### Quantitative futility

**Figure 1:**
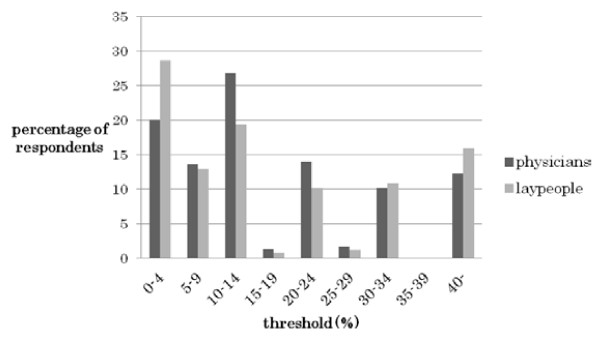
Distribution of thresholds of quantitative futility (235 physicians and 756 laypeople).

One hundred sixty-six (41.4%) physicians and 380 (33.5%) laypeople did not identify a threshold that indicated quantitative futility. The thresholds chosen varied widely in both groups: 0–80% in physicians and 0–95% in laypeople (Figure 1). Median and mode were both 10% in both groups. Among the respondents who identified a threshold, 79 physicians (33.6%) and 314 laypeople (41.5%) chose thresholds under 10%. Seventeen physicians (7.2%) and 125 laypeople (16.5%) identified a threshold of 0%.

### Provision of futile treatment

Among 401 physician-respondents, 88.3 percent had experienced providing futile treatment: 159 (39.7%) several times a year, 95 (23.7%) several times a month, 66 (16.5%) several times a week, and 34 (8.5%) almost every day. Request for the treatment from the patient or patient’s family (1, 2), to satisfy the patient (7), lack of public standards about judging futility or forgoing treatment (13, 14), avoidance of legal issues (16), and communication issues (5, 6) were main reasons for providing treatments that physician-respondents judged futile (Table 3).

## Discussion

The basis of the medical futility issue is disagreement between healthcare professionals and patients: patients or their families request treatments judged futile by healthcare professionals. Our survey largely focused on the evaluation of this disagreement by exploring attitude differences between physicians and laypeople. Although our survey has limitations, especially in sampling, our findings still offer useful information for understanding the issue. The main outcome of our survey is that laypeople are more supportive of providing potentially futile treatments than physicians, consistent with the central problem described above. Including consideration of the factors for judging a certain treatment futile (Table 2), we mainly discuss the difference in likelihood of supporting the provision of potentially futile treatments.

### The gap in the importance of medical information

“Healthcare worker’s inadequate explanation about futility of the treatment” (5) and “Patient’s insufficient understanding about futility of the treatment” (6) were main reasons physician-respondents gave for providing treatments judged futile (Table 3). Communication between healthcare workers and patients is a principal dimension of medical futility [2,3,6,8,12]. Its improvement, including the provision of medical information, will contribute to the resolution of the issue. Physicians are in an optimal position to evaluate the empirical and physiological aspects of futility. Four aspects of medical information gaps between physicians and patients in Japan have been discussed: knowledge of medicine, outlook after intervention, experience with similar cases, and views on the event (patients are subjective, whereas physicians are objective) [16]. By eliminating anxiety, fear and doubts of patients, and helping them make rational judgments, healthcare workers should be able to address these gaps. However, even if these gaps are removed, there is doubt about agreement between both sides.

In Vignette 1 of our questionnaire, physician-respondents stressed the professional opinion of the doctors more than laypeople (77.5% versus 44.6%, *P* = 0.000, Table 1). Also, physicians placed more emphasis on “Physical harm (side effect or complication) of the treatment” (16) and “Theoretical medical appropriateness of providing the treatment” (17) as factors for judging a certain treatment futile (Table 2). Because adverse effects of treatments may cause the death of debilitated patients, physicians may be nervous about providing such treatments. The principle of non-maleficence may allow physicians to forego them [4]. With the development of evidence-based medicine, physicians are urged to practice not only based on theory or rationale but more importantly based on actual physiological effectiveness, and are reluctant to deviate from them. The findings of our survey suggest that such professional standards may not be accepted by patients and their families. Even if patients have sufficient information because of efforts by healthcare workers, judgments may differ if there are gaps in degree of importance of this medical information.

### The involvement of the patient’s family in medical decision making

“Request for the treatment from the patient’s family” (2) was considered to be an important factor for judging futility by laypeople rather than physicians (Table 2). The involvement of family members of Japanese patients in medical decision-making has been illustrated in previous literature. In a recent survey, Japanese patients themselves wished for their family’s involvement in medical decision-making and patients’ family members wished to take part [17]. Collectivism (individuals consistently conceptualized as part of a larger group and expected to subordinate personal goals to those of the group) and Confucianism (in which harmony and obedience are viewed as virtues) are underlying characteristics of the family’s strong influence on medical decision-making in Japan [9]. A triadic relationship is emphasized in this model of decision making, or rather, patients may sometimes withhold their own interests and follow their family members’ opinion, even when it differs from their own. Furthermore, in a public survey in 2008, attitudes of laypeople toward life-prolonging treatments differed between their own end-of-life conditions and their family members’: such treatments were wished for family members more than for themselves [10]. In another survey regarding percutaneous endoscopic gastrostomy for severely demented elderly (which was raised as a futile treatment by some participants in our preliminary survey [11]), maintaining the family’s peace of mind was a trigger for the procedure [18]. Sentiments and thoughts of family members often act in the direction of life prolongation of the end-of-life patient. Japanese physicians must be careful of the patient’s family’s wishes and consider the psychological benefits generated by the treatments. The family’s strong involvement in medical decision-making and their request for treatment, which was valued by laypeople in this questionnaire (Table 2), potentially become triggers for providing treatments that bring the patient little quantitative or qualitative benefit, and are sometimes harmful. Physicians who sincerely adopt a patient-centered approach must be reluctant to provide such treatments.

### Quality of life and life-prolongation

“Therapeutic effect for quality of life (QOL) of the patient” (9) was stressed as a factor for judging a certain treatment futile by physicians (Table 2). Physicians’ priority of QOL seems to also be a reason for the differences between groups (Table 1). Some guidelines regarding end-of-life care have been established in Japan in the last few years that include mention of “meaningless” or “non-beneficial” life-prolonging treatments. Though medical futility is not explained in these guidelines, it is supposed to be one factor for their establishment. The guidelines established by the Japan Medical Association mentions that QOL should be respected and is one basis for forgoing life-prolonging treatment, as well as wishes of the patient and human dignity [14]. The interpretation is that forgoing active treatments for end-of-life patients based on improvement or maintenance of the patient’s QOL is more beneficial than continuation of treatments that may potentially harm the patient. Including the spread of palliative care in recent years, it is supposed that many physicians have come to include QOL, which justifies forgoing treatments judged futile.

However, the opposite form of end-of-life care, which continues active treatments to the last breath of the patient, has also been employed in Japanese healthcare settings. It is thought that this form is based on Shintoism, which governs Japanese spirituality and established the Japanese view of death. In Shintoism, death is a curse. Abhorrence of death still resides in the mind of the Japanese and facilitates reluctance towards any termination of life [19]. Some participants in a survey of family members of deceased cancer patients wished for continuation of active treatments, and the authors discussed that the QOL of the patient included the continuation of such treatment as long as possible [20]. In our survey, physicians emphasized patient conditions of brain death and severe dementia (in which the QOL of the patients are thought to be objectively low) significantly more than laypeople (Table 1). Asai et al., in their analysis of Japanese public opinion regarding brain death and the definition of death in the revised Organ Transplant Act, discussed that some Japanese think that medical doctors have a duty to make an all-out effort to save a person’s life, regardless of the condition, and that patients in irreversible comatose states should be valued and offered medical care unstintingly [21]. Even if the patient cannot be aware of the benefit from medical treatments, there are likely to be some people who affirm the provision of active or life-prolonging treatments for such patients. Forgoing treatments based on consideration of the patient’s QOL may be unacceptable for proponents of such perspectives. It is supposed that even today conventional views about end-of-life, such as the value of prolonging life and abhorrence of death steadfastly remain. Healthcare workers’ excessive emphasis on QOL in end-of-life care conflicts potentially leads to disagreement with patients holding such views. Also, psychological impact on patients and their families (12, 13 in Table 3) was emphasized by laypeople in the third section of the questionnaire. This is interpreted as the treatments themselves providing significant psychological benefits for people who value providing life-prolonging or aggressive treatments as much as possible, no matter what the effect on the body and QOL of the patient is. Such benefit, which is not substantive but collateral, may be very important for the patient and their family. Forgoing such treatment may cause the patient and their family to feel abandoned [6].

### Arbitrariness of quantitative futility

In our survey, both groups chose “Likelihood of recovery or cure due to the treatment” (15) as a factor for judging a certain treatment futile (Table 2). This indicates that the quantitative aspect of futility (numerical probability that an act will produce the desired physiological effect) is important for judging futility. The recent rise of evidence-based medicine (EBM) may be very useful for judging futility [8]. In a survey conducted by McCrary and colleagues that asked US physicians about quantitative assessments of futility in 1991, there was a lack of agreement regarding the threshold that defines futility, [22] similar to the results of our survey in which the threshold showed wide variation in both groups (Figure 1). Additionally, 41% of physicians and 34% of laypeople did not identify a threshold. Many respondents commented that quantifying was very difficult and that the threshold was case-dependent (data not shown). These findings demonstrate the arbitrariness of the quantitative futility concept. The decision that treatment is quantitatively futile involves value choices [7]. On the other hand, very low thresholds (0–1%) were identified more frequently by laypeople than by physicians (27.0% versus 17.9%, Figure 1). This is consistent with situations in which patients who believe in miraculous successes request provision of treatments that healthcare workers are reluctant to provide (based on their futility judgment).

In contrast, what about cases of patients who do not prefer very low thresholds of quantitative futility? In such cases, should healthcare workers follow the professional demand to continue treatment as long as there are chances of recovery or to make full effort even if no chance exists? If they follow it, they may be forced to provide treatments that the patients do not want. Establishing a consistent threshold of quantitative futility may cause stress for both physicians and patients. McCrary and colleagues discussed that physicians’ unilateral determination of futility based on quantitative assessments raises the potential abuse of discretion and casts doubt on the justice of such a decision-making process because of its lack of agreement [22]. In addition, we found that patients are also unable to reach a consensus on the numerical fixation of quantitative futility because of its arbitrariness.

### Physicians’ experiences of futile treatment in daily clinical settings

In our survey, 88% of physicians reported that they had experienced providing treatments that they judged futile. This indicates that futility, which has been seldom discussed in Japan, occurs in daily clinical settings and has not been adequately addressed. The lack of a system that provides guidance on forgoing treatments and avoiding legal repercussions was the main reason for providing such treatments (Table 3). These results are consistent with reports from other countries [12]. Although medical futility is often portrayed as a disagreement between physicians and patients, our results suggest that mutual agreement between the two parties is insufficient. In Japan, there were some cases in which removing mechanical ventilation from end-stage patients was considered to equate with breaking the law, regardless of mutual agreement. There has been turmoil about withdrawal of life-prolonging treatment that may result in inappropriate accusations against well-meaning doctors, put unnecessary burdens on dying patients and their families, mislead the mass media and public, and trouble police and prosecutors [23]. As expected, communication was another major reason for providing futile treatments (Table 3). Taking the results at face value, an improved decision-making process and preparation of guidelines and laws regarding end-of-life care may resolve the futility issue. This survey was not designed to cover measures for resolving the futility issue, but to demonstrate differences in value judgments between physicians and laypeople. The differences indicate that professional standards of healthcare workers cannot be applied to patients in some situations. A large interventional survey discussed that enhancing opportunities for communication is inadequate, and that social commitment and proactive, forceful measures might be needed [24]. Guidelines and laws addressing medical futility may help healthcare workers, but what measures can obtain mutual agreement on treatment goals and moreover, a social consensus?

### Limitations

There may be a social desirability bias because the questionnaire asked about forgoing of treatments, which could potentially cause patients’ deaths and medical conflicts. Though all respondents responded anonymously, some might not have answered with their true intent.

Sampling bias must also be considered. We sampled physician-respondents, who answered paper-and-pencil questionnaires, via personal networks and we did not adjust their distributions (age, sex, practice term, specialty and working facility). Results of this study cannot represent the overall awareness of Japanese physicians on medical futility. On the other hand, laypeople were sampled and completed the questionnaire through a web-based method. In Japan, where the Internet penetration rate was 78.2% in 2010, internet surveys have headed the list of marketing research methods since 2005. Although this method has some advantages, including improved data quality, rapid response and a lower likelihood for social desirability bias, there is a coverage bias. Attitudes of Internet non-users and laypeople over 70 years old were not reflected in this survey. Also, this method potentially yields different results compared with off-line methods [25]. Though the questionnaire answered by both groups had the same content, it can’t strictly be said that both groups were compared under the same conditions.

In this survey, the completion rate of the laypeople-respondents who answered via the Internet questionnaire was 7%. Response rates for web-based questionnaires are generally lower than for postal questionnaires [25]. If participants are sampled randomly from a large number of potential responders, a low response rate is not necessarily a problem [26]. Our consigned research company (Cross Marketing Inc.) sampled randomly from 1.35 million registered panelists. The fact that there were panelists who failed to review the questionnaire within the delivery term and the difficulty of understanding the content of the questionnaire were considered reasons for the low completion rate. The survey was designed so that the number of deliveries is set based on the delivery term and estimated number of responses (1134 responses in about 2 days for the present survey). A substantial numbers of e-mails were delivered due to the fact that many panelists fail to connect to the Internet within the specified term. McGee pointed out technical difficulties of studies assessing patients’ reactions to futility: outcome variables are difficult to define and questionnaires are difficult to validate [27]. Though we used vignettes for clarification of the survey theme, added brief explanations of medical terminologies and asked closed-ended questions, all laypeople-respondents might not have understood completely. In fact, some respondents commented as such. However, 886 (78.1%) of laypeople-respondents freely commented about their actual experiences including end-of-life care, life-prolonging treatments, decision-making, care for the elderly, health expenditures, and so on. This may suggest that many respondents answered through their deliberation.

On the other hand, many layperson-panelists who failed to complete the questionnaire might also have had difficulty in understanding medical information. Because less educated people may tend to have unrealistic expectations to medicine in actual clinical setting, the low completion rate of this survey may suggest that the current results could have underestimated the difference of attitudes between laypeople and physicians towards the issue of medical futility.

## Conclusion

The results of this survey suggest that patients are more affirmative in providing potentially futile treatments than physicians in Japan. The difference of attitudes towards such treatments can be explained by the gap in the importance of medical information, the conventional family-oriented approach in medical decision-making and healthcare workers’ relatively-excessive emphasis on the QOL of the patient. Also, although the quantitative aspect of medical futility is useful to judge a certain treatment futile, we would hardly reach a consensus on its numerical fixation because of its arbitrariness.

## Competing interests

The authors declare that they have no competing interests.

## Authors’ contribution

All authors (YK, AA and SB) designed and conducted the questionnaire survey. YK analyzed the data with feedback from AA. All authors participated in writing the manuscript and approved the final version.

## Funding

This research was supported by “A Research Project to Develop Patient-Healthcare Professional Relationships for ‘Health Care Thinking Together’,” Basic Research (A) #20249035, Grant-in-Aid for Scientific Research from the Japan Society for the Promotion of Science, 2008–2010.

## Ethical approval

The present study was approved by the ethics committee of Kumamoto University Graduate School of Medical Science in July 2010 (receipt No. 366).

## Pre-publication history

The pre-publication history for this paper can be accessed here:

http://www.biomedcentral.com/1472-6939/13/7/prepub
